# The physiological and thermal responses of military personnel undertaking a military exercise in Kenya

**DOI:** 10.1186/2046-7648-4-S1-A19

**Published:** 2015-09-14

**Authors:** Sophie Britland, Simon Delves, Mike Stacey, Joanne L Fallowfield

**Affiliations:** 1 Institute of Naval Medicine, Gosport, Hampshire, UK; 2 Royal Centre for Defence Medicine, Birmingham, UK and Section of Anaesthetics, Imperial College London UK

## Introduction

Undertaking operational patrolling activity in hot environments, carrying heavy loads and wearing personal protective equipment (PPE) presents a significant physiological challenge to Service personnel. Planned research trials involving volunteers exercising in the heat observe an important volunteer withdrawal criterion of attainment of a core body temperature of 39.5 °C. However, trained populations (marathon runners) have been shown to sustain exercise with core temperatures in excess of 40 °C with no recognised adverse health effects [1]. Thus, it could be hypothesised that military personnel with a good level of physical fitness might similarly sustain higher than usual core temperatures, albeit while wearing impermeable PPE. The aim of the present study was to measure the *actual *thermal response of military personnel while performing operationally specific exercises in the most arduous phase of a military exercise in a hot-dry environment.

## Methods

A cohort of n = 28 male personnel from 3^rd ^Battalion the Parachute Regiment volunteered to participate in this study (MODREC 465/Gen/13). Physical characteristics; age, 25 (5) years; height, 1.80 (0.06) m; body mass, 81.1 (9.5) kg; sub-group maximal oxygen uptake, 60.7 (6.3) mL.kg.min^-1 ^[2]. Physiological and thermal measures were made over two consecutive 24 hour periods (Day 1 and Day 2) during the military Exercise ASKARI STORM. This is a live firing tactical training activity in Kenya delivered by the British Army Training Unit Kenya. Each 24 hour period contained a combat scenario (Live Firing 1 and 2). Volunteers were instrumented with a heart rate monitor (Polar Team 2, Polar, Finland) *in situ *prior to the beginning of exercise on each day. Additionally, volunteers swallowed ingestible core temperature pills (VitalSense, Mini Mitter Company Inc, USA) at 12 hour intervals throughout the observation period.

## Results

Mean WBGT for Day 1 and Day 2 were 25.9 (4.2) and 24.2 (4.9) °C, respectively. Throughout Day 1 and Day 2 mean core temperature remained below 38.5 °C (Day 1, 37.4 (0.4) °C; Day 2, 37.1 (0.4) °C and although there was a high degree of variation between individuals, all volunteers remained below the critical T_core _of 39.5 °C. At the onset of each Live Firing scenario, T_core _rose (Live Firing 1, 1.0 (0.6) °C.hr^-1^; Live Firing 2, 0.3 (0.4) °C). However, after this initial rise, T_core _either decreased (Live Firing 1) or attained a plateau (Live Firing 2). Rate of rise was lower when considered over the duration of each scenario (Live Firing 1, 0.2 (0.3) °C.hr^-1^; Live Firing 2, 0.1 (0.4) °C.hr^-1^). Due to technical difficulties, heart rate measurements were only collected during Live Firing 1, where the majority of time (60 (11)%) was spent in the moderate heart rate zone (40-59 %HRmax), whilst 20 (9)% was spent in the hard zone (60-84 %HRmax).

## Discussion

Despite volunteers undertaking challenging military-specific work in the heat, core temperature was successfully regulated over both a prolonged period (Day 1 and 2) and during an intense combat scenario (Live Firing 1 and 2). This successful maintenance was most likely a result of good aerobic fitness and positive behavioural responses to the activity and environment (i.e. adequate rest and venting opportunities such as removing helmet and loosening PPE).

## Conclusion

Throughout the 2 day observation period core temperature was elevated but successfully maintained below 39.5 °C. In this well-trained population, the risk of attaining a critical core temperature was low in the environmental conditions and at the intensities undertaken here.
